# The rhetoric of cancer

**DOI:** 10.1007/s10552-014-0428-9

**Published:** 2014-07-11

**Authors:** Matthew Trendowski

**Affiliations:** Department of Biology, Syracuse University, 107 College Place, Syracuse, NY 13244 USA

**Keywords:** Cancer, Awareness and prevention, Carcinogens, Epidemiology, Risk groups

## Abstract

**Background:**

Research involving the discovery of novel anticancer drugs and treatments hold precedence among the general public. However, investigating the etiology and epidemiology of malignancies can have a significant effect on reducing the prevalence of cancer in society. Understanding risk factors that drive neoplastic development can provide educated individuals the opportunity to avoid such catalysts.

**Methods:**

Literature searches were conducted on prominent magazine and newspaper sources to analyze the accuracy and relevance the material had toward cancer prevention. Additionally, two professionals involved in oncology were interviewed to gain a more personal view of the population’s knowledge on cancer awareness and prevention.

**Results:**

The lack of attention paid to the understanding of cancer and its subsequent prevention has resulted in fundamental misconceptions that facilitate the development of neoplastic growths.

**Conclusions:**

Addressing the lack of attention paid to cancer awareness and prevention through proper education can have a significant effect on limiting the impact cancer has on society.

## Introduction

There is no avoiding it. If one lives long enough, he or she will eventually be impacted by the debilitating malignancy known as cancer. Even if the individual is lucky enough to elude cancer, it is likely that a close friend or family member will be stricken by a malignant neoplasm. The prevalence of cancer suggests that public awareness should be significantly heightened in order for everyday citizens to be well informed and capable of making conscious decisions in regard to their health. Perhaps the most fundamental of these concepts is that cancer is not a single disease, but rather a collection of related diseases which all share the common feature of aberrant cell proliferation [1]. As crucial as this knowledge is for understanding the very basis of cancer, it is not being emphasized in most grade school curriculums. It is not even being sufficiently expressed in popular media outlets, such as television or magazines where essential medical knowledge is conveyed [2]. Ideas that are fundamental to oncologists are somehow not being passed onto the general population where they would serve the most benefit.

There is an alarming discrepancy between the extent of information medical professionals have toward cancer, and the actual knowledge that is passed onto the general population [2]. However, this subsequently begs the question, why is there such a discrepancy? Why is this information not being sufficiently broadcasted to the general population in public awareness campaigns where it would serve the most benefit? Therefore, the purpose of this commentary is to highlight major misconceptions the general public has about cancer and then propose solutions to alleviate these discrepancies. If such issues related to the rhetoric of cancer are understood, reform can be brought to both the medical profession and public health awareness, so that this crucial information is not lost in translation.

## Professional insight

The input of experienced professionals proved to be invaluable for understanding how potentially threatening misconceptions of cancer awareness and prevention could have developed, as their expertise shed light on critical health issues that often seem to be left by the way side. Like most scientists in the field, Dr. Thomas Fondy, a cancer biology professor at Syracuse University (Syracuse, NY) felt as though his work has barely scratched the surface in terms of fully understanding the complexities of cancer pathology. The unpredictability of malignant neoplasms makes every discovery and development of potential treatments a monumental achievement. However, whenever Dr. Fondy tells someone about his research, the single most common question he is asked is if he or any of his colleagues are close to discovering a cure for cancer. Unfortunately, it seems as though the general population is out of touch with reality when it comes to understanding how difficult it actually is to inhibit and eventually eliminate neoplastic growths.

Misconceived notions are all too common among frightened patients, as they are uncertain of what their diagnosis actually means. When asked to describe the most common misconceptions patients have after their initial diagnosis, Dr. Stephen Graziano, an oncologist at Upstate University Hospital (Syracuse, NY), indicated that many individuals have no idea of what their expected prognosis actually is. There are many individuals who believe that the recent advances in science will surely provide a way to eradicate their cancer before it becomes a serious threat. This often stems from the fact that most individuals still view cancer as a single disease and believe a miracle cure will soon be in reach. Conversely, there are a substantial amount of patients who believe that their diagnosis is inherently a death sentence, even though the death rate for many cancers is actually decreasing [1]. Individuals who are diagnosed with the same form and severity of cancer can therefore have a completely different perception of treatment options that are available.

One of the most troubling misconceptions Dr. Graziano has noticed through the years is the lack of belief people have in using routine diagnostic procedures to detect cancers while they are still in a manageable state. Many of these individuals believe that they are too young or that the procedure is too expensive for serious consideration. Unbeknownst to them, they might have a genetic predisposition to a specific cancer, putting them at a much higher risk than the general population. This is frequently seen in individuals who suffer from familial adenomatous polyposis, an inherited condition that results in uncontrolled polyp growth within the large intestine [3]. Since colon cancer is often derived from polyps that progress into malignant growths, these individuals are likely to develop life-threatening symptoms at an age much earlier than is expected. If these individuals do not adhere to routine polyp screening, they will almost certainly be diagnosed with colon cancer by the time they reach forty. Such patients usually have a dim prognosis, as the cancer has often metastasized to other tissues by the time individuals begin to present symptoms [3]. Routine screening can often make the difference between being diagnosed with a removable benign tumor, or a malignant neoplastic growth that has corroded many vital organs, severely reducing the chance of survival.

## Lack of attention paid to preventative measures

Important information regarding preventative measures toward cancer is often left out of popular media outlets. After analyzing the top search results for cancer in the websites for *Time*, *Newsweek*, *AARP*, *Reader’s Digest*, *New York Times*, *Los Angeles Times* and *USA Today*, which are some of the most widely distributed magazines and newspapers in the United States, several alarming trends emerged. The majority of these articles focused on stories more geared for grabbing the reader’s attention than providing insightful information on cancer, such as harrowing accounts of individual cancer battles or the controversy of applied research techniques to uncover potential cures. Articles that focused on specific types of cancer tended to focus exclusively on the most common types of neoplasms, such as those found in the breast or colon, and in many cases never mentioned preventative measures that could be taken against the malignancies. Unfortunately, the website that did provide the most information on cancer and epidemiological patterns was for *AARP*, a magazine traditionally reserved for senior citizens. This is a disconcerting trend, as cancer is more often than not a protracted disease, meaning it can take years before causative agents end up inducing a malignant tumor [4]. Therefore, information regarding environmental factors associated with cancer should be publicized in newsprints that are advertised for younger audiences, as they still have time to make effective use of preventative measures.

## Misconceptions result in flawed perceptions

With the inadequate media coverage of fundamental cancer basics being apparent, it is no surprize that several alarming misconceptions have developed among the public. While there have been numerous polls developed in recent years that concern the state of public knowledge toward cancer, the most comprehensive to date was a study done by the American Cancer Society [5]. Results from the survey revealed distressing misconceptions that appeared to be common among the American public. Almost 40 % of the adults who responded to the poll believed that living in a polluted city posed a greater risk for lung cancer than smoking a pack of cigarettes a day. The belief is completely erroneous, as the study points out that between 80 and 90 % of all deaths attributed to lung cancer is the direct result of smoking on a consistent basis. Multitudes of studies stemming back decades have warned of tobacco smoke’s cancer inducing capabilities. There was even a study published as early as 1795 that found a correlation between pipe smokers and an increased likelihood for lip cancers [6].

The other extremely disconcerting misconception that the American Cancer Society study revealed was that many individuals do not understand the significance of healthy living as a young adult. According to the survey results, an estimated 25 % of participants believed that lifestyle choices made as a young adult had almost no bearing on cancer incidence later in life. Despite the fact that both lung and skin cancer have been linked to habits that are developed early in life, there is still a considerable amount of individuals who feel that health decisions made as a young adult have no significant influence on their likelihood of contracting cancer. The potentially devastating implications of this misconception cannot be overexpressed as cancers of the lung and skin have some of the highest known mortality rates when found in their malignant state. Lung cancer that has progressed into the metastatic stage has a survival rate of just 3.7 % over a 5-year period. Patients diagnosed with metastatic melanoma do not fare much better with a 15 % survival rate over the same period [7]. When concepts as fundamental to cancer as the link between lifestyle choices in young adults and subsequent cancer risk are being misinterpreted by the public, the gap of knowledge between medical professionals and everyday citizens becomes even more apparent.

## Lost in translation

While it would seem inherent for raising cancer awareness, portraying relevant cancer information to the general population in a readily accessible format does not appear to be consistently followed. In fact, many articles available online appear to be left in sophisticated scientific contexts that most individuals would find difficult to fully comprehend. This disparity was epitomized in a study conducted by researchers at Loyola University Medical Center who analyzed 62 popular websites that commented on prostate cancer and associated treatment options that are available for afflicted patients [8]. Articles were read multiple times and subsequently evaluated to determine what grade level of reading comprehension would be necessary for an adequate understanding of the conveyed information. Although the National Institutes of Health (NIH) recommends that information publicized on patient health should be written between a 4th and 6th grade level to ensure that concepts are properly understood, the investigation revealed that almost all of the sites published articles that required a 12th grade or higher level of reading comprehension [8]. The level of reading capability required is well beyond the scope of many individuals as almost a third of the US population reads at an 8th grade level or lower [8]. While this statistic may be somewhat skewed as it is unclear if children were taken into account, the expectancy of 12th grade reading comprehension is still beyond the means of many Americans who are unable to adequately comprehend material written at this level.

The findings of the Loyola University Medical Center study are extremely disconcerting when coupled with the fact that prostate cancer is the single most common neoplasm found in men, resulting in more than 28 % of new diagnoses every year [9]. This suggests there are thousands of men being diagnosed with prostate cancer every year that do not understand their affliction and even more importantly are unaware of the available treatment options. Even when patients are given a list of potential treatments, they are typically unaware of what the medication regimen or diagnostic procedure will entail or how it will actually inhibit neoplastic growth [8]. While individuals are usually provided with literature that details important aspects of the suggested treatment, the material is again written in a sophisticated style that many patients are simply unable to fully comprehend.

Unfortunately, the dilemma is a direct reflection of the apparent failure to transfer vital information regarding cancer awareness and prevention to the public where it would have the greatest impact. By not properly translating the complexities of cancer into clear, readily accessible formats, many individuals are left unaware of the threat that these malignancies present. The inexcusable gap of knowledge between medical professionals and everyday citizens is fostering an environment in which individuals unknowingly increase their risk of contracting cancer and then are left incapable of making thoughtful decisions when they do receive the diagnosis. This haphazard approach has and will continue to put the lives of countless individuals at risk unless affirmative measures are taken to correct the undeniable discrepancy.

## Conclusion

The analysis of public awareness toward cancer uncovered several unsettling misconceived notions that derive from fundamental misunderstandings of such malignancies. Cancer is not a single disease; it is a collection of related diseases that have distinct phenotypes and invasive properties, making the development of effective treatments extremely arduous. Although cancer is fundamentally a genetic disease that is spurred on by uncontrollable aberrant mutations within an individual’s DNA, these mutations can be significantly reduced if certain lifestyle choices are avoided. However, some concepts relating to cancer pathology are not as easy to comprehend and often require trained professionals to translate the potentially lifesaving information into a form that is readily understandable. Unfortunately, deciphering such complex ideas into a style that requires little to no prior scientific knowledge can prove to be exceeding difficult. Textual resources that are written to benefit the everyday citizen are often portrayed in a sophisticated language that requires substantial education to properly comprehend. Even worse, popular media outlets that have the potential to reach countless individuals often fail to comment on critical issues associated with cancer. Instead, focus is usually directed toward dramatic accounts of individual cancer battles or groundbreaking cancer research that might one day result in the miracle cure the general public longs for. Although these stories are usually thought provoking and informative, they ultimately distract everyday citizens from understanding vital concepts, such as correlations between lifestyle choices and specific types of cancer, which would serve a more immediate benefit.

Cancer has a prodigious socioeconomic impact on society, as evidenced by the estimated $125 billion spent on cancer care each year in the United States alone [10]. Therefore, increasing educational efforts to lower the rate of cancer incidence could be an efficient method to reduce such an overwhelming healthcare burden. Individuals who are aware of effective preventative measures are more likely to incorporate such beneficial lifestyle choices into their daily routine, which in theory can significantly reduce the incidence rate of cancer each year. Less cancer diagnoses would inherently result in less money being spent on treating afflicted patients. More importantly, it would substantially decrease the staggering percentage of the population that develop cancer and eventually die from resulting complications. After all, keeping patients healthy so they can live a meaningful and productive life is the ultimate goal of medicine. If measures are taken to reduce the devastating burden cancer elicits, the benefits will reverberate for generations to come, as individuals will have the capability to take matters into their own hands and end the battle with cancer before it begins.

## References

[1] Weinberg RA (2013). The biology of cancer.

[2] Redmond K (2007). Promoting better quality media coverage of cancer. Nat Clin Pract Oncol.

[3] Strate LL, Syngal S (2005). Hereditary colorectal cancer syndromes. Cancer Causes Control.

[4] Alberts B, Johnson A, Lewis J (2007). Molecular biology of the cell.

[5] Park A (2007) Top five cancer misconceptions. Time.com

[6] Proctor RN (2001). Tobacco and the global lung cancer epidemic. Nat Rev.

[7] National Cancer Institute (2014) Cancer Statistics 2014. http://www.cancer.gov/statistics

[8] Ellimoottil C, Polcari A, Kadlec A, Gupta G (2012). Readability of websites containing information about prostate cancer options. J Urol.

[9] Siegel R, Ma J, Zou Z, Jemal A (2014). Cancer Statistics, 2014. CA Cancer J Clin.

[10] Farina KL (2012) The economics of cancer care in the United States. Am J Manag Care 18:Web 22468873

